# N2GNet tracks gait performance from subthalamic neural signals in Parkinson’s disease

**DOI:** 10.1038/s41746-024-01364-6

**Published:** 2025-01-04

**Authors:** Jin Woo Choi, Chuyi Cui, Kevin B. Wilkins, Helen M. Bronte-Stewart

**Affiliations:** 1 Department of Neurology and Neurological Sciences, Stanford University School of Medicine, Stanford, CA USA; 2 Department of Neurosurgery, Stanford University School of Medicine, Stanford, CA USA

**Keywords:** Biomedical engineering, Machine learning, Neurophysiology

## Abstract

Adaptive deep brain stimulation (DBS) provides individualized therapy for people with Parkinson’s disease (PWP) by adjusting the stimulation in real-time using neural signals that reflect their motor state. Current algorithms, however, utilize condensed and manually selected neural features which may result in a less robust and biased therapy. In this study, we propose Neural-to-Gait Neural network (N2GNet), a novel deep learning-based regression model capable of tracking real-time gait performance from subthalamic nucleus local field potentials (STN LFPs). The LFP data were acquired when eighteen PWP performed stepping in place, and the ground reaction forces were measured to track their weight shifts representing gait performance. By exhibiting a stronger correlation with weight shifts compared to the higher-correlation beta power from the two leads and outperforming other evaluated model designs, N2GNet effectively leverages a comprehensive frequency band, not limited to the beta range, to track gait performance solely from STN LFPs.

## Introduction

Gait has been noted as one of the heavily researched areas for movement disorders in Parkinson’s disease (PD), as its related symptoms including impairment, slowness of stepping, or freezing may cause devastating incidents such as falling^1–3^. One of the possible ways to alleviate these symptoms is through deep brain stimulation (DBS), an implantable system that delivers electrical stimulation to specific brain regions such as subthalamic nucleus (STN)^4–6^. By providing a consistent level of stimulation determined by the clinicians to people with PD (PWP) throughout their daily activities, DBS has been adopted in various situations to reduce medication usage and treat motor symptoms^7^.

The advancement of DBS allowing sensing capabilities from its leads has paved the way for the therapy to be a closed-loop system, where DBS can adjust its parameters automatically based on sensed neural activity^8,9^. This recently developed approach is known as adaptive DBS, which adjusts the amount of stimulation in real-time with respect to the motor performance and reduces the exposure of unnecessary amounts of stimulation^10,11^. One of the common neural signals that adaptive DBS utilize is the local field potential (LFP) recorded from the STNs that reflect the motor state of PWP. Specifically, neural oscillatory activity within the beta frequency band (13–36 Hz) has been known to be associated with movement, including changes in bradykinesia and freezing of gait (FOG)^12–16^, serving as a useful indicator for adjusting stimulation levels in real-time. Growing evidence has highlighted that adaptive DBS using these biomarkers was effective in alleviating motor symptoms while delivering less stimulation compared to conventional open-loop DBS^17,18^.

Various adaptive DBS algorithms have been proposed to speculate patient’s movement performance from beta-related biomarkers and modulate stimulation accordingly. For instance, beta power had been used in single and dual-threshold algorithms, where the stimulation was adjusted depending on one or two clinically defined beta power thresholds, respectively^19–21^. Beta burst durations have also been investigated for their feasibility to drive the stimulation, with its consideration that prolonged beta bursts are more associated with motor disability and gait impairment than short bursts^22,23^. Despite these advancements, using beta power or burst durations with threshold-based algorithms possess some limitations. Due to the nature of neural oscillatory patterns being different between individuals and even within individuals over time, the algorithms using these condensed neural signal features may result in less reliable therapy^24–26^. In particular, these algorithms employ low-complexity representation of signals that may also be affected by other components that do not correlate with real-time movements, such as cognitive impairment, plastic effects, and severity of motor symptoms^24,27,28^. Relying on these low-complexity features that can potentially be influenced by multiple causes may lead to degraded performance of the algorithm. These methods also use handcrafted parameters identified through visualization to determine which of these compressed neural features should be considered, which may fail to capture detailed neural characteristics associated with real-time gait of an individual. Such approaches are thus prone to becoming less reliable, more subjective, and biased therapies due to their low complexity^29^.

In this paper, we introduce Neural-to-Gait Neural network (N2GNet), a deep learning-based data-driven approach capable of predicting gait performance in real-time relying on LFP signals from left and right STNs. Considering that unsupported gait may raise safety concerns such as falls, the LFP data was acquired while participants performed stepping in place (SIP) while being harnessed, and the ground reaction forces from the left and right legs were measured through the corresponding force plates to alternatively assess gait performance^30^. To have the model learn features automatically from high-complexity LFP signals, our deep learning architecture was designed to extract features directly from band-pass filtered LFP signals, which would be associated with the amount of weight shifts performed by participants during the SIP task. With the extraction of relative features that aims to learn relative changes between pairs of oscillatory components from the LFPs, our model is designed to enhance gait prediction performance beyond merely utilizing direct oscillatory characteristics of the signals.

## Results

### Participant demographics

A brief overview of the dominant symptom for each of the eighteen participants is shown in Table 1. Participants were divided into two groups depending on their dominant symptoms, a tremor dominant (TD) group and an akinetic rigid (AR) group, resulting in nine participants per group.

**Table 1:** Participant demographics and data information

Participants	Symptoms	Train			Validation			Test		
		Visit Mo.	Length (s)	Value range	Visit Mo.	Length(s)	Value range	Visit Mo.	Length (s)	Value range
1	TD	6	190	(0.0–0.95)	9	193	(0.0–0.9)	12	182	(0.0–1.0)
2	TD	39	193	(0.0–0.99)	42	184	(0.0–1.0)	52	158	(0.0–0.95)
3	TD	12	176	(0.0–1.0)	21	174	(0.0–0.86)	33	172	(0.0–0.64)
4	TD	1	169	(0.01–1.0)	3	206	(0.01–0.98)	6	187	(0.02–0.72)
5	TD	3	179	(0.0–1.0)	16	181	(0.0–0.94)	32	138	(0.0–0.99)
6	TD	1	160	(0.0–0.97)	3	166	(0.0–0.91)	6	174	(0.01–1.0)
7	TD	IP	165	(0.0–1.0)	9	108	(0.0–0.83)	27	214	(0.0–0.78)
8	TD	6	203	(0.0–1.0)	21	219	(0.01–0.07)	27	117	(0.01–0.07)
9	TD	30	157	(0.0–0.99)	33	149	(0.0–1.0)	36	158	(0.0–0.98)
10	AR	6	166	(0.0–0.97)	12	171	(0.0–1.0)	33	158	(0.0–0.94)
11	AR	21	147	(0.0–0.88)	27	137	(0.0–1.0)	33	211	(0.0–0.93)
12	AR	12	155	(0.0–0.77)	28	195	(0.0–0.79)	32	183	(0.0–1.0)
13	AR	3	163	(0.0–1.0)	6	176	(0.0–0.98)	12	180	(0.0–0.95)
14	AR	6	166	(0.0–1.0)	9	183	(0.0–0.91)	12	185	(0.0–0.63)
15	AR	34	205	(0.0–1.0)	37	161	(0.0–1.0)	41	154	(0.0–0.96)
16	AR	31	151	(0.0–1.0)	34	161	(0.0–1.0)	38	140	(0.0–0.96)
17	AR	4	140	(0.0–1.0)	6	152	(0.0–0.98)	12	139	(0.0–0.79)
18	AR	3	177	(0.0–0.78)	6	186	(0.0–1.0)	9	194	(0.0–0.97)

The value ranges for labels are reported as (minimum value − maximum value) in each set.

*TD* tremor dominant, *AR* akinetic rigid.

The months the visits took place after the initial programming (IP) of DBS, the duration of task recordings in seconds, and the range of weight shift data labels from each task are also shown for training, validation, and testing datasets in Table 1. The IP visits, which were held for the initial activation of the DBS system, took place a month after the implantation of the DBS leads. The MDS-UPDRS (Movement Disorder Society-Unified Parkinson’s Disease Rating Scale) III scores for when participants were off medication and OFF stimulation, which were evaluated within three months of each visit, are also available in Supplementary Table 1 for reference.

### N2GNet performance on neural-to-gait translation

**Fig. 1:**
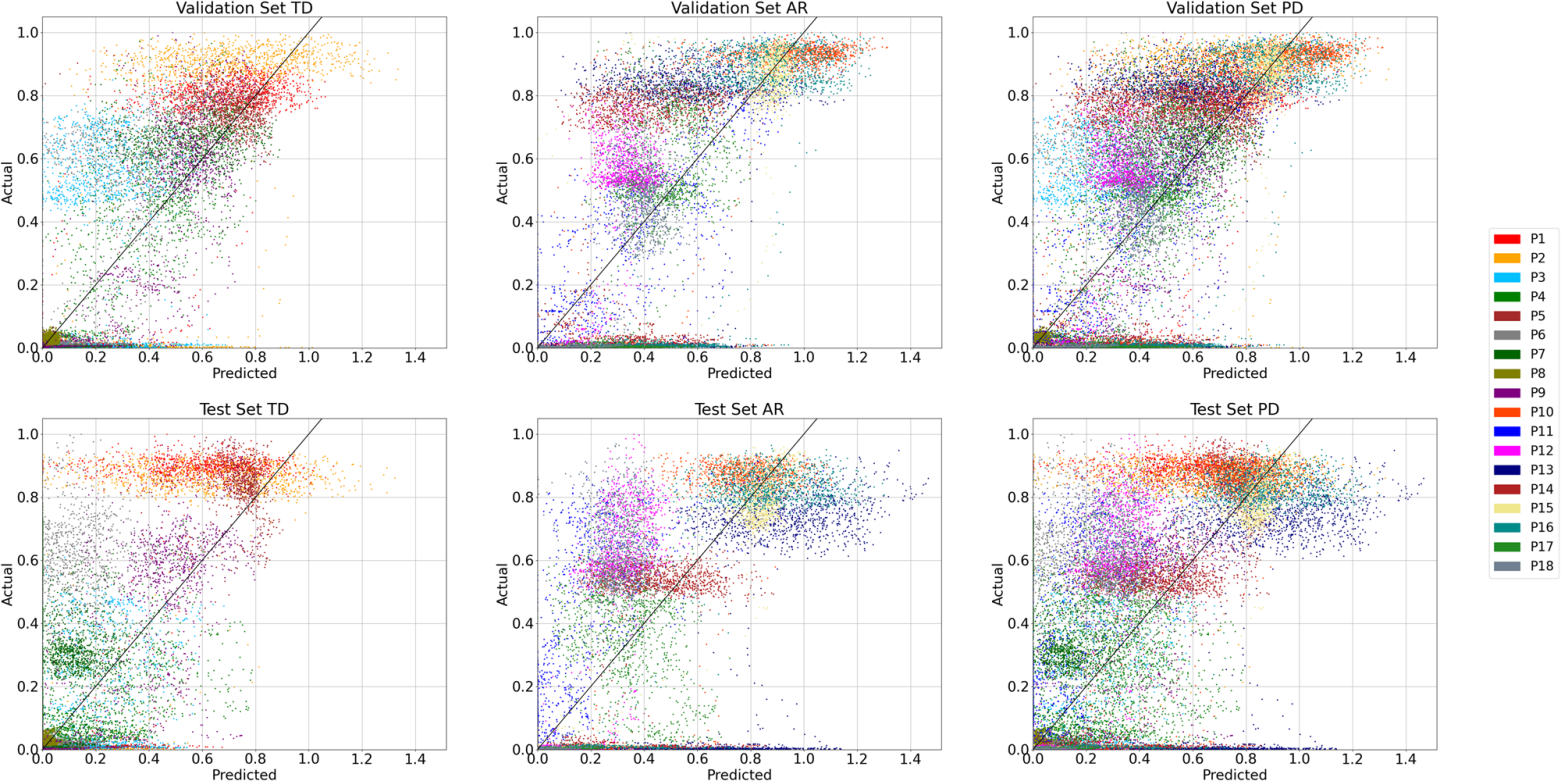
Performance of N2GNet’s predictions for TD group, AR group, and for all PD participants. The *x*-axis indicates the predicted values from our proposed model and the *y*-axis indicates the actual values, which are the weight shift values computed from the force plates.

The performance of N2GNet, which inputs 5-second LFP data to predict the last 2 seconds of weight shift measure during participants’ repeated steps on the two force plates, was investigated for each participant using the three SIP task datasets acquired across three different visits. Figure 1 shows the spread of N2GNet’s results for the validation and test sets retrieved from the midpoint and latest visits, respectively, after training the model with the training dataset from the earliest visit. The results for TD group participants showed a mean ± standard deviation of 0.132 ± 0.075 for mean absolute error (MAE) and 0.041 ± 0.034 for mean squared error (MSE) with validation datasets, and an average of 0.157 ± 0.083 for MAE and 0.059 ± 0.051 for MSE from test datasets, showing 0.025 and 0.018 increments in the average MAE and MSE, respectively. AR group participants also had an increase in error rates from validation to test datasets, with 0.004 average MAE increments from 0.186 ± 0.088 for validation sets to 0.19 ± 0.093 for test sets, and with 0.009 increments in average MSE from 0.063 ± 0.042 for validation sets to 0.072 ± 0.067 for test sets. No significant differences were observed between the two groups for both MAE and MSE from both validation (MAE with *U* = 27.0, *unadj* − *p* = 0.258 and MSE with *U* = 28.0, *unadj* − *p* = 0.297, Mann–Whitney *U*-test) and test sets (MAE with *U* = 31.0, *unadj* − *p* = 0.436 and MSE with *U* = 36.0, *unadj* − *p* = 0.73, Mann–Whitney *U*-test).

The overall N2GNet performance with all PD participants using MAE was 0.174 ± 0.089 for the test datasets, which was 0.015 higher than the average error rate using validation datasets (0.159 ± 0.086). The average MSE was 0.065 ± 0.06 in test datasets, which was also greater by 0.013 than the average MSE from validation datasets, which was 0.052 ± 0.04.

### Correlation analysis with N2GNet and with beta power

**Fig. 2:**
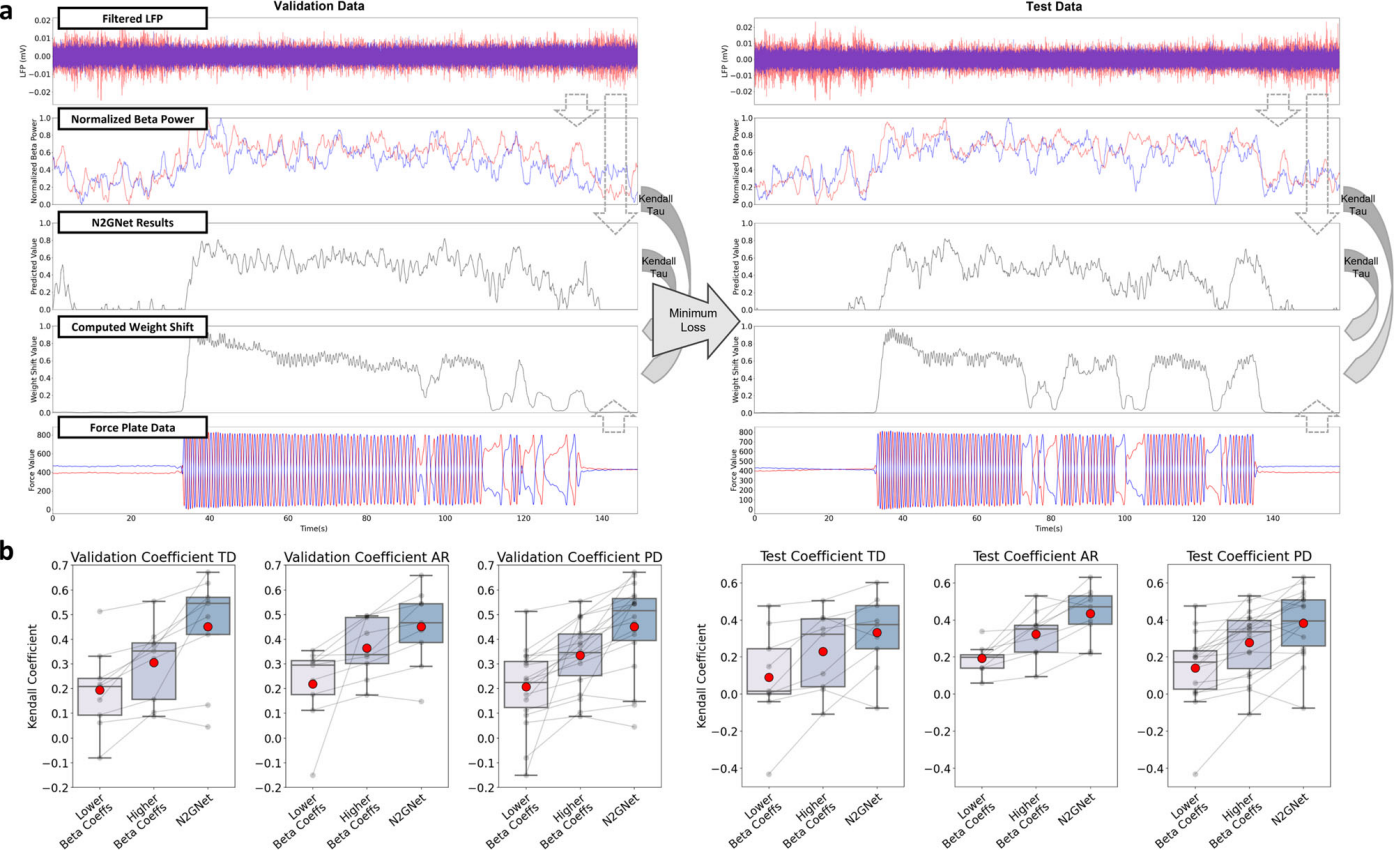
Kendall tau correlation analysis. **a** Example Kendall tau correlation comparisons demonstrating the procedures, where the two beta power results acquired separately from the two LFP signals and the prediction results from the N2GNet was referred to the weight shifts computed from the force plates to calculate correlation coefficients. **b** Comparisons of correlation results from validation and test sets for TD group, AR group, and for all PD participants. The x-axis, from left to right, indicates the lower coefficients out of the two beta powers measured from each participant, the higher coefficients of the two, and the coefficients computed with N2GNet results. The gray dots indicate correlation coefficients from each participant, and the dots in red represent the mean value. The boxplots represent first quartile, median, and third quartile for lower, middle, and upper lines in the boxes, respectively, whereas the whiskers represent 1.5 times the IQR extending above the first quartile and below the third quartile.

Kendall tau coefficients were measured to evaluate and compare N2GNet’s performance and beta power for reflecting weight shifts. The correlation results using Kendall tau coefficient between beta power measures and weight shifts, and between predicted values from N2GNet and weight shifts are shown in Fig. 2. As demonstrated in Fig. 2a, average beta power measures over a 2-second interval corresponding to the weight shifts were computed separately for each lead, and the correlation between each beta power measures and the weight shifts was compared to the correlation between N2GNet’s results and weight shifts.

The comparisons between the beta power of lower coefficients, the beta power of higher coefficients, and the coefficients with N2GNet’s predictions show that stronger correlations were exhibited with N2GNet’s predictions than with the beta power from either of the two leads (Fig. 2b). The average Kendall tau coefficient for the TD group from validation sets using N2GNet was 0.45 ± 0.206, which was greater than the average of 0.305 ± 0.147 from the beta power of higher coefficients. N2GNet also exhibited greater Kendall correlation on test sets with 0.33 ± 0.195 compared to the higher-coefficient beta power of 0.229 ± 0.203. As for the AR group, N2GNet’s predictions had higher correlation with the weight shift than the beta power in both validation (0.45 ± 0.148 for N2GNet and 0.364 ± 0.111 for higher-coefficient beta power) and test sets (0.434 ± 0.134 for N2GNet and 0.323 ± 0.123 for higher-coefficient beta power).

Overall with PD participants, N2GNet’s predictions were to have significantly higher correlation with weight shifts compared to the beta power with higher coefficients from the two leads. The prediction results from the model exhibited average correlation coefficient of 0.45 ± 0.179 for validation datasets and 0.382 ± 0.175 for test sets, whereas the beta power with higher correlation exhibited an average coefficient of 0.335 ± 0.134 and 0.276 ± 0.174 for validation and test datasets, respectively (*W* = 5.0, *unadj* − *p* = 7.629e-5 for validation sets and *W* = 20.0, *unadj* − *p* = 2.808e-3 for test sets, Wilcoxon signed-rank test). Other possible frequency bands of interest were also additionally explored for reference and can be seen in Supplementary Fig. 1.

### N2GNet model ablation study

**Fig. 3:**
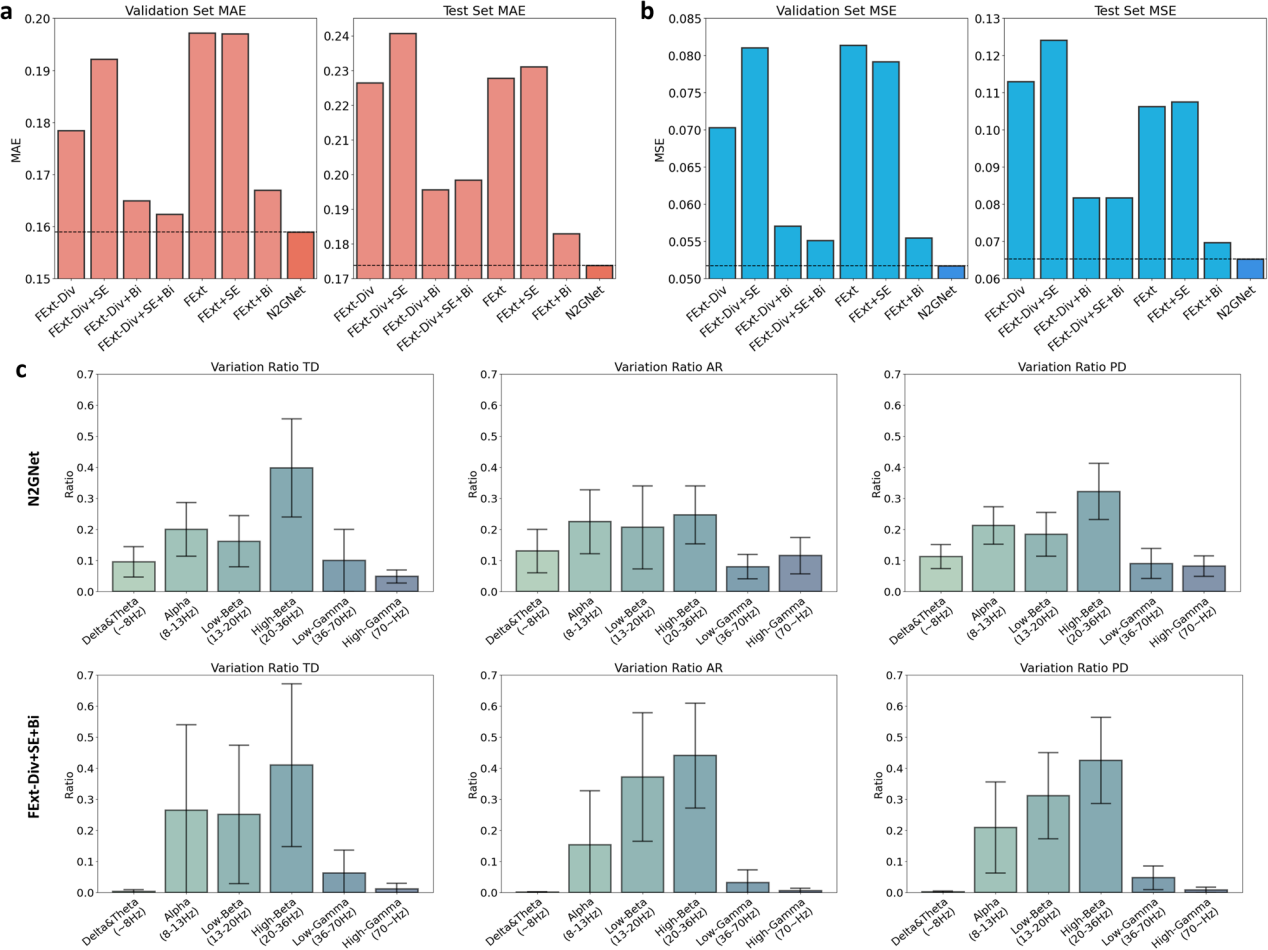
Model ablation study results and analysis. The average error rates using **a** MAE and **b** MSE on validation and test sets for eight different model designs derived from our proposed model framework. **c** Variation ratio results quantifying the impact the data of certain frequency bands of interest affected on the output of the model. Both N2GNet and N2GNet without element-wise division in the feature extraction block (FExt−Div+SE+Bi) were analyzed to explore the effect of considering relative oscillatory features in our model. The error bars represent 95% confidence intervals.

Our N2GNet architecture consists of four different blocks: a feature extraction block (FExt), a feature squeeze and excitation block (SE), a bi-directional long short-term memory block (Bi), and a regression block, where the feature extraction block includes an element-wise division process (Div). To explore how each block composing our N2GNet affected the prediction performance, a model ablation study was held by eliminating possible block combinations from our N2GNet (details of the model designs can be seen in Supplementary Fig. 2). The results with our participant data and with seven other possible model designs showed that our N2GNet was able to outperform other models in both MAE and MSE error rates. As can be seen from MAE results in Fig. 3a, N2GNet had a lower average error rate compared to when bi-directional long short-term memory (LSTM) block was removed (FExt+SE, validation error: 0.197, test error: 0.231), in the absence of feature squeeze and excitation block (FExt+Bi, validation error: 0.167, test error: 0.183), and in the absence of considering relative features (FExt−Div+SE+Bi, validation error: 0.162, test error: 0.198). Similarly for the average MSE (Fig. 3b), N2GNet had the least error rate on both validation data and test data compared to the absence of bi-directional LSTM block (mean validation error: 0.079, mean test error: 0.108), in the absence of feature squeeze and excitation block (mean validation error: 0.055, mean test error: 0.07), and in the absence of considering relative features (mean validation error: 0.055, mean test error: 0.082). Other possible combinations also exhibited higher error rates than our N2GNet in terms of both MAE and MSE.

Figure 3c further shows variation ratios with respect to different frequency bands for TD group, AR group, and for all PD participants. The analysis was held on our proposed N2GNet and also on the model similar to N2GNet but without the element-wise division process (FExt−Div+SE+Bi) in order to see the effect of considering relative oscillatory features in our model. Results from the model that did not consider relative features exhibited high variation ratios from alpha and beta bands for both TD group (alpha: 0.265 [95% CI: −0.01 to 0.539], low-beta: 0.25 [95% CI: 0.027–0.473], high-beta: 0.409 [95% CI: 0.147–0.672]) and AR group (alpha: 0.152 [95% CI: −0.021 to 0.326], low-beta: 0.371 [95% CI: 0.164–0.578], high-beta: 0.44 [95% CI: 0.271–0.609]) compared to other frequency bands of interest. Similarly, N2GNet also exhibited high ratios in these bands for TD group (alpha: 0.199 [95% CI: 0.113–0.286], low-beta: 0.161 [95% CI: 0.079–0.244], high-beta: 0.397 [95% CI: 0.239–0.555]) and AR group (alpha: 0.224 [95% CI: 0.121–0.327], low-beta: 0.206 [95% CI: 0.072–0.339], high-beta: 0.246 [95% CI: 0.153–0.339]). Apart from the alpha and beta bands, N2GNet showed relatively higher ratio on gamma frequency bands (PD group, low-gamma: 0.089 [95% CI: 0.041–0.138], high-gamma: 0.081 [95% CI: 0.049–0.114]) compared to the model without relative features (PD group, low-gamma: 0.047 [95% CI: 0.009–0.085], high-gamma: 0.008 [95% CI: −0.001 to 0.017]).

We additionally conducted analysis with the models trained and validated with the LFP data band-pass filtered with only the beta band apart from the original signals which were band-pass filtered between 8 to 100 Hz range (Supplementary Fig. 3a, b). When the model was trained and tested with beta-filtered LFP signals, models that included relative features elicited an increased MAE and MSE error rates in validation datasets compared to when original signals were used, resulting in other models to outperform N2GNet with validation datasets for MAE (FExt−Div+Bi: 0.167, FExt−Div+SE+Bi: 0.16, N2GNet: 0.17) and MSE (FExt−Div+Bi: 0.057, FExt−Div+SE+Bi: 0.054, N2GNet: 0.058). Yet, N2GNet still outperformed other models on the test sets with its average MAE and MSE of 0.2 and 0.075, respectively. The variation ratio results in Supplementary Fig. 3c further show that the model that excluded relative features was mostly influenced by beta bands, whereas N2GNet still utilized other frequency bands besides the beta range despite the use of beta band-pass filtered signals for its training and evaluation.

## Discussion

The objective of our regression model is to extract neural features that reflect real-time gait of people with PD, solely relying on STN LFPs that do not require additional sensors and can be retrieved directly from the two DBS leads. Our results of mapping neural recordings with the weight shifts measured during SIP tasks exhibited a mean absolute error of 0.174 ± 0.089 and mean squared error of 0.065 ± 0.06 with our proposed N2GNet model using the dataset with continuous labels ranging from 0 to 1. To provide justification for each block composing our model, we further performed a model ablation study employing different deep learning structures that were derived from our proposed architecture. The results demonstrated that our proposed model, in its full structure, was able to achieve the lowest error rates in both MAE and MSE compared to other model designs evaluated in this study. These error rate results indicate that our N2GNet’s complete structure not only had the lowest quantitative differences towards weight shifts but also exhibited less larger-scale errors and outliers. Moreover, the results from Kendall tau correlation analysis elaborate that our model was able to effectively utilize LFPs from the two leads, with its predicted outcomes having greater correlation with the weight shifts from the SIP task compared to either of the beta power measures from the two leads. Most importantly, while our variation ratio from the models marked a strong emphasis around the beta range indicating its importance in predicting gait as mentioned in previous studies^12,15^, our additional analysis involving models trained and evaluated with beta-filtered signals along with these results further highlights the importance of taking a wide range of frequency bands into account and not limiting to the beta range. By mapping LFP signals directly into the weight shift values acquired during the SIP task, our N2GNet provides insights into future adaptive DBS algorithms that utilize data-driven deep learning methods to predict real-time gait performance, which would be utilized for real-time adjustment of stimulation parameters.

One of the important factors that was considered in designing our N2GNet architecture was the aperiodic component from neural signals. Previous studies had addressed that these aperiodic changes can occur due to age-related cognitive impairments^27^ and the severity of motor symptoms^28^. Furthermore, aperiodic changes could be observed in PWP throughout 18 months of their visits after the implantation of DBS^24^, which do not necessarily reflect direct movements of PWP. These aperiodic activity changes may not only degrade the performance of low-complexity algorithms that utilize handcrafted thresholds, but may also crucially influence machine learning approaches with designs that are prone to overfitting. The use of relative oscillatory features computed through the element-wise division process, which was held in our feature extraction block, was thus employed to have the model consider not only the direct oscillatory characteristics of the signals but also the relative changes in major oscillatory features in relation to other oscillatory components. This matter is designed with inspiration from previous studies that normalized beta LFP signals with signals from the gamma band, which are more stabilized and less prone to artifacts^31,32^. Throughout our results, we were able to confirm that the N2GNet benefited from this approach by exhibiting lower error rates compared to when the division procedure was removed.

In line with the improvement of performance by jointly considering relative oscillatory features, the use of such characteristics necessitated further development of an analysis to explore which frequency bands had greater influence on the model during training. We supposed that simply utilizing the error rates derived by inputting signals filtered to specific frequencies of interest would be inappropriate, as this approach would not take into account shifts in the model’s prediction resulting from the fixed parameters trained with wider-band original signals. While using more narrowly filtered signals as input to the model that was trained with wider signals indicates that the training set and input data were from overlapping yet still different frequency domains, the possibility of shifts in the distribution of electrophysiological data may occur^33–35^, making direct error rate measurement from these filtered signals unreliable for investigating their importance. This issue would be more critical for our N2GNet, where the feature extraction block also utilizes relative changes between pairs of extracted oscillatory patterns beyond focusing solely on direct oscillatory features, possibly being more prone to shifts in the outcomes of the feature extraction block. Thus, inspired by previous literature that investigated the association of different frequency bands on the classification result by correlating signals with multiple frequency bands with how much they have affected the model’s output^36^, we developed and utilized the variation ratio presented in our study to gain insights into how much these different frequency bands of interest affected our model.

In this regard, our analysis highlights the drawbacks of relying mostly on beta signals for designing adaptive DBS algorithms. The results of comparing the variation ratios of different frequency bands of interest showed that N2GNet exhibited a comparatively even distribution of ratios across different bands and achieved lower error rates than FExt-Div+SE+Bi, the model that did not consider relative features (Fig. 3c). Furthermore, comparing Fig. 3a, b and Supplementary Fig. 3a, b shows that all our models showed increased error rates on the test sets when beta-filtered LFPs were used for training and testing the model, compared to when the original signals of 8–100 Hz band ranges were used. These results support our claim that relying solely on the beta band may weaken the model’s performance. It is also worth noting that the models containing the element-wise division process had smaller error rate differences between the validation and test sets compared to the models without division. Such observations imply that including relative features through our division process, along with considerations of other frequency bands beyond the beta band, provided greater stability in prediction performance over time, as there existed time gaps between the acquisition of validation and test data. With the fact that the only difference between the models in terms of their architectures is the existence and absence of element-wise division within the feature extraction block, the results in our study demonstrates that taking the entire spectrum of signals into account and considering relative oscillations between different frequency bands may enhance the algorithm’s performance, especially when the algorithm needs to maintain its performance over an extended period.

Surprisingly throughout our model ablation study and the variation ratio analysis from Fig. 3c and Supplementary Fig. 3c, we discovered that the N2GNet was able to utilize signals outside the band-pass filtered range, while the other model without element-wise division exhibited its ratio most entirely within the filtered range. We suspect that the cause of such results is the band-pass filter's inability to completely eliminate signals outside the desired band ranges^37,38^. Our N2GNet’s utilization of relative features through element-wise division tends to consider the ratio between two different oscillatory features rather than the direct scale, indicating that the model’s relative feature computation would require additional oscillatory feature to act as reference for the original oscillatory feature. This may have led our N2GNet to consider signals outside of filtered band ranges during model training, even though their scales were greatly reduced through filtering. To that extent, the aforementioned limitation of band-pass filtering may have also caused minor frequency overlaps between adjacent bands during our variation ratio analysis, even after filtering, which could have slightly affected the results.

In addition to the aforementioned phenomenon identified through the variation ratio analysis, it is also important to stress that our variation ratio measure serves as an indication of which frequency band signals caused more variability to the model’s prediction outcomes, which implies that the model’s architecture can change the results. Exhibiting higher variance in a specific frequency band does not always signify a stronger theoretical correlation with gait, as our variation ratios are completely determined from the perspective of the trained model itself. This should be reminded even stronger for our N2GNet which utilize relative oscillatory features, as the model has the potential to use frequency ranges that are unrelated to gait as a reference feature. Thus, our variation ratio results have limitations and should be interpreted carefully along with consideration of the model’s overall architecture. Rather than as a thorough interpretational method, our variance analysis should serve as an indication that demonstrated improvements in prediction performance by taking into account wide frequency ranges.

Our N2GNet exhibited prediction performance without significant difference between the TD and AR groups in our study, suggesting that the model may be robust for different subtypes of PD. This may be an interesting result as TD typically elicits less changes in beta than AR during movement^39,40^, while both TD and AR can still exhibit gait impairment and FOG^41^. Note that one of the factors that caused the average MAE and MSE from the validation and test datasets to be relatively smaller in the TD group was due to one of the participants exhibiting freezes throughout the entire task from the visits corresponding to the validation and test datasets (Participant 8). The participant did not experience major freezes during the task in the visit that was used as training data, resulting in low MAE and MSE results for that particular participant.

Through our study, we aimed to address the potentials of using deep learning-based methods for predicting real-time gait of PWP solely with STN LFPs, which can further be extended into adaptive DBS algorithms in the near future. Contrary to previous algorithms that utilize compressed features such as beta power or beta burst duration, the ultimate goal of our work is to have features automatically learned within the model, providing optimal and personalized therapy for each individual patient. Unlike current parameter determination procedures which involve individuals performing multiple movement tasks and clinicians repeatedly observing, evaluating movements, and tuning necessary parameters throughout summarized information without taking detailed features into account, our N2GNet is geared towards automatically adjusting the parameters with only a few SIP recording trials. To also resemble practical usage of our proposed model, we evaluated our model in a way that the training, validation, and test datasets were determined in a chronological order, with each dataset containing a single SIP recording session from a visit.

While this is merely a step towards bringing a deep learning-based method to adaptive DBS, limitations exist when it comes to the practical usage of N2GNet in real-life adaptive DBS systems, and future works can be conveyed to enhance our algorithm. Our model is trained to translate LFP signals into weight shifts that resemble gait, however, these weight shifts may not always correlate with the ideal amount of stimulation needed for actual walking. For instance, it is possible that certain PD-related symptoms, such as shuffling with its light but frequent weight shifts, may rather increase the amount of weight shifts and result in a decrease of stimulation when the model is trained with weight shifts as labels. Although we aimed to filter such possibilities by applying a low-pass filter to the force plate data at 2 Hz, further investigation of whether such a pre-processing method would effectively eliminate these instances should be conducted. The nature of LFP signals having aperiodic components serves as another limitation, as our method of extracting relative oscillatory features still does not make the model completely independent from the aperiodic neural activity. Evaluating the model with a constrained sample size can be another limitation in our study, as increasing the sample size with additional data from other participants in each of the PD subtypes could facilitate a more rigorous analysis of the used models. Improvements in prediction performance can further be held and designing lighter models that reduce computational cost should also be conveyed in order to embed the algorithm into the DBS system. Lastly, and most importantly, our current N2GNet architecture does not consider any possible influences from stimulation. The model should consider both direct contamination of signals from stimulation and indirect physiological consequences that could alter oscillatory features in any way. Further works to enhance the model’s robustness to stimulation can be carried out in advance for the practical application of N2GNet in real-life DBS systems.

In this study, we developed a novel deep learning-based model that relies only on local field potentials from the subthalamic nucleus to predict real-time gait performance of people with Parkinson’s disease. Our N2GNet achieved the lowest error rate in prediction by using relative oscillatory features, which were obtained by simply adding an element-wise division process that resulted in considering a broader range of signal frequency bands. Compared to other model designs from our model ablation study, N2GNet exhibited greater stability in its performance across both validation and test datasets, which were composed of data acquired considerably after than those from training datasets. Our study not only shows the potential of applying deep learning for adaptive DBS algorithms in keeping track of real-time gait performance with local field potentials but also emphasizes the benefits of taking overall frequency ranges, beyond just utilizing beta bands known to be associated with movement, into account for gait prediction.

## Methods

### Participants

The data from eighteen participants who were diagnosed with PD, implanted with bilateral STN DBS leads connected to an implanted investigative neurostimulator (Activa PC + S, Medtronic, PLC), and met the established criteria for dataset formation were used in our study. Participants were instructed to perform the experiment in the off-medication state. This involved stopping long-acting dopamine agonists at least 48 hours, dopamine agonists and controlled release carbidopa/levodopa at least 24 hours, and short acting medication at least 12 hours before testing. The study was performed in accordance with the Declaration of Helsinki. All participants gave written consent prior to the study. The study was approved by the Food and Drug Administration with an Investigational Device Exemption (G130186) and by the Stanford University Institutional Review Board (Approval Number: 25916 and 30880).

### Experimental protocol

Participants were involved in multiple visits after the implantation of DBS, where the visits for each participant were held at least a month apart. Per each visit, participants were asked to be engaged in the SIP task once without stimulation.

**Fig. 4:**
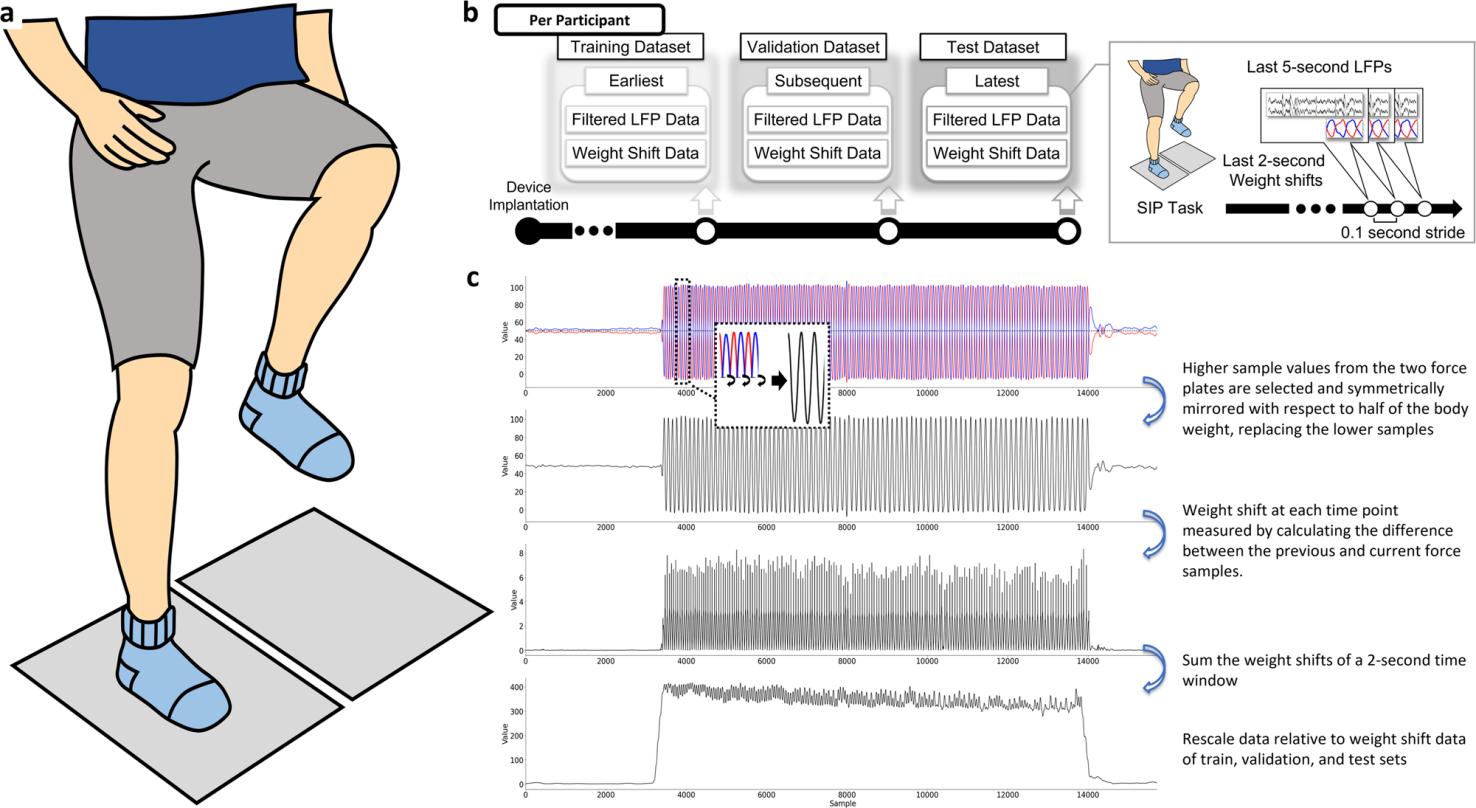
Experiment protocol for N2GNet. **a** Data acquisition phase where the LFPs from the two leads were measured while participants were to perform SIP on the two force plates. **b** An illustration of the experimental diagram regarding the formation of datasets for model evaluation. A single SIP task was performed during each visit, and the data from three different visits was assigned to training, validation, and testing datasets in chronological order. **c** Procedure for weight shift calculation from the force plate data. The two force plate data samples from each time point were merged by selecting the sample with greater value, and samples from one of the force plates were mirrored symmetrically with respect to the value representing half the participant’s weight. Changes in the resulting merged samples were then calculated over a 2-second time window to retrieve the weight shift measure of the corresponding time interval.

For each SIP task, participants performed a single round of SIP after DBS had been turned OFF for at least 15 minutes. To prevent any falls, participants wore safety jackets that were securely harnessed to the force plate system. In the beginning of the task, participants were first instructed to stand on two force plates that measure ground reaction force on each foot and were asked to remain as motionless as possible. Participants were then given a start cue and were expected to alternatively lift their legs at their own pace for approximately 100 seconds, simulating the movement of walking but remaining within the force plates (Fig. 4a). Lastly, participants were given a stop cue and were instructed to stop the movement after. The LFP signals from left and right STNs were recorded simultaneously with the measures from the two force plates.

### Data acquisition and dataset formation criteria

Ground reaction forces were measured using either Neurocom (Neurocom Inc., Clackamas, OR, USA) or Bertec (Bertec Corporation, Columbus, OH, USA) at sampling rates of 100 Hz and 1000 Hz, respectively, with their two force plates. For the ground reaction forces measured at a 1000 Hz sampling rate, the data were downsampled into 100 Hz to maintain consistency across data. The LFP signals were initially sampled with a sampling rate of 422 Hz and were downsampled to a 211 Hz sampling rate to train the deep learning model with lighter complexity. A pre-processing step was held on the retrieved force plate data by applying a low-pass filter of 2 Hz, in order to reduce jerky noises that are less likely to be related to gait. The LFP signals were also band-pass filtered using a fourth-order infinite impulse response (IIR) filter with a frequency range of 8–100 Hz in order to include a comprehensive range of frequency bands that may contain information associated with movement^42,43^.

As participants were involved in multiple visits performing the SIP task, we established criteria for the formation of datasets that were applied to each participant. Given that deep learning models require training, validation, and test datasets for training and evaluating the model, only the data from participants who completed the task at least three times on separate occasions were selected for this study. Specifically, data from the earliest visit was used as a training set, data from the subsequent visit as a validation set, and data from the latest visit was used as a test set for the model, resulting in a use of data from three different visits for each participant (Fig. 4b). This usage of visits in chronological order was done to limit the amount of data used for training the model while reflecting the actual usage scenario, where the model would be trained on past data while continuously receiving input of the most recent data for predictions. The datasets from each participant were formed in a way that their recording contacts remained consistent across the three visits. For participants who had their implantable pulse generators re-implanted between their visits, only the data collected before the re-implantation were included to maintain consistency of signal quality across datasets.

The LFP data from each visit was visually inspected prior to the data formation to exclude visits that contained excessive noise or artifacts, which critically distorted the overall signals across all frequencies. As our model aimed to function independently without relying on other complex algorithms, no additional methods for removing specific types of artifacts were applied to the LFP signals after band-pass filtering. Thus, the possibility still remains that the data may have contained minor artifacts, such as those associated with cardiac activity or gait^44,45^.

### Gait quantification through weight shifts

Previous work has demonstrated that abnormalities in gait initiation may lead to a reduced lateral shift of body weight in PWP^46^. In addition, it has also been addressed that the movement amplitude during lateral weight shifts is smaller preceding unsuccessful steps^47^, and is correlated with bradykinesia and postural instability^48^. In addition to these aforementioned factors, as the SIP task requires participants to perform repeated lateral weight shifts, we utilized weight shift measures derived from the force plates to reflect continuous SIP performance (Fig. 4c).

Prior to measuring weight shifts, the force plate data obtained from each task was rescaled by dividing each data sample by the participant’s weight, which was also retrieved from the two force plates when the participants remained motionless. This adjustment was done prior to quantifying weight shifts such that our measurement would be robust to variations in the participants’ weight over different visits.

As participants distribute their weight over the two force plates when both feet are in contact, the measurements from the force plates are inversely proportional to each other. However, this correlation may not always hold true when one of the feet is off contact during the SIP task. Previous work had demonstrated that there may be cases where changes in force on the contacted force plate may still occur while participants tend to shift back towards the other force plate that is not yet in contact^30^. To take these patterns into consideration, the ground force samples from the plates were merged into a single value per time point by selecting the higher sample of the two, which disregarded samples with lower force measures including those of when the feet was off contact. Subsequently, samples from one of the force plates were mirrored symmetrically with respect to the value that signified half the participant's weight. Thus, samples having higher values would indicate more weight being placed on one force plate, while samples having lower value would represent more weight being distributed toward the other force plate. The change in force resulting from this merged outcome was quantified over a two-second time window to represent the amount of weight shift during that specific time interval.

Considering that the task performance of each individual participant varies between visits and the performance also varies among participants, the data was normalized within each participant by mapping the maximum weight shift value of the two-second time window out of the three visits into 1, with a completely motionless state set as 0.

### Deep learning model architecture

**Fig. 5:**
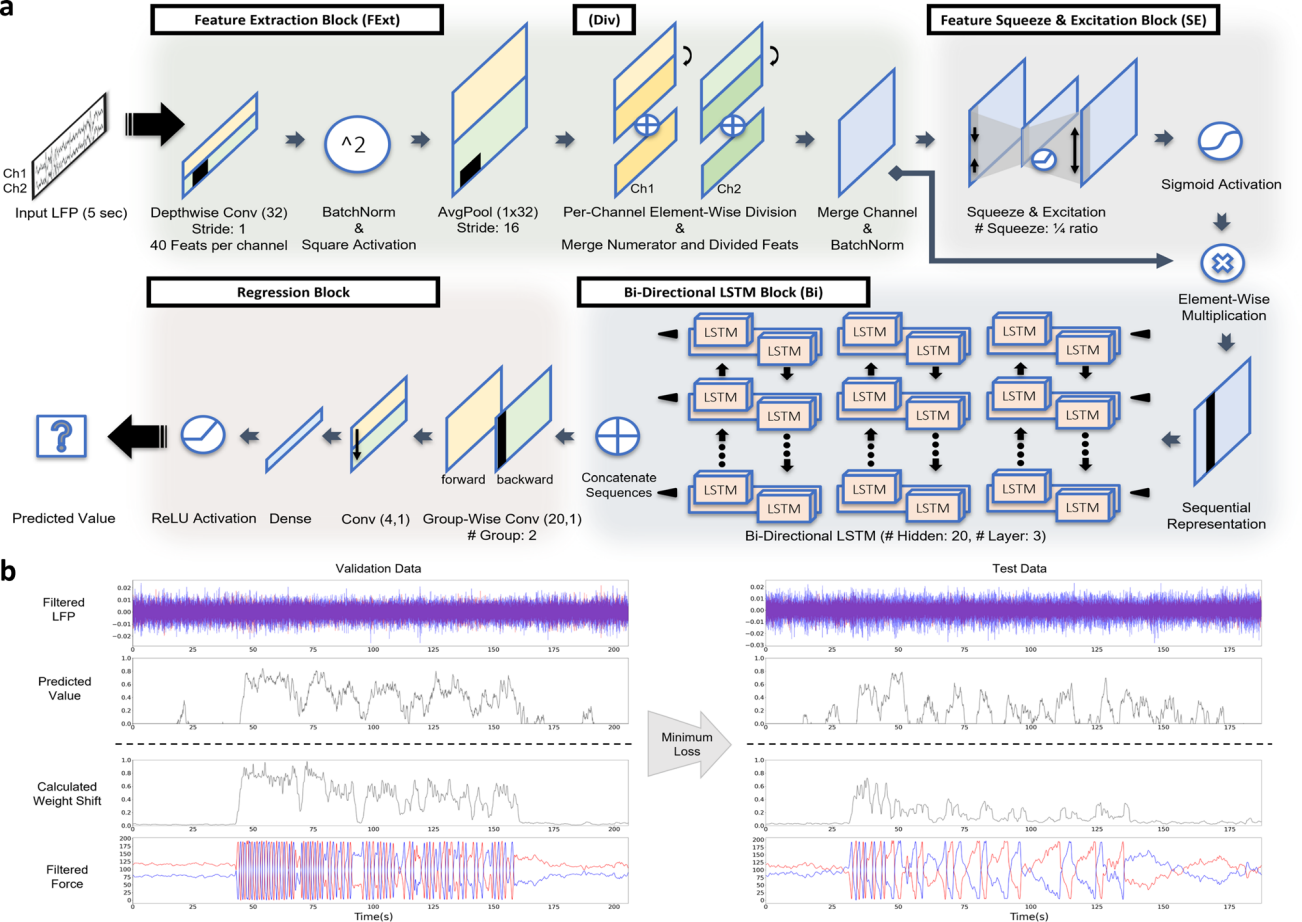
N2GNet model architecture. **a** The model takes in 5-second window LFP signals from the two leads and produces a single outcome representing gait performance. **b** Example of N2GNet results when continuously inputting LFP signals retrieved from the two leads. The model trained on the epoch with minimum l1 error rate from the validation dataset was chosen to assess its performance on the test dataset.

The architecture of our model is composed of four main blocks in a sequential manner: the feature extraction block, the feature squeeze and excitation block, the bi-directional LSTM block, and the regression block as shown in Fig. 5a. The model processes the band-pass filtered LFP signals to generate a single value outcome representing the predicted weight shift that the participant would have exhibited.

- Feature extraction block: It has been shown that convolutional neural networks (CNNs) are capable of extracting oscillatory features^36,49^. With inspiration from these previous approaches, our feature extraction block utilized 1-dimensional CNN with batch normalization, square activation function and average pooling to imitate the power measure computation from the inputted two band-pass filtered LFP signals. A 1-dimensional CNN was used in a depthwise manner, where each channel includes features from LFP of a single lead, such that the LFPs from the bilateral STNs are learned separately. Given the presence of aperiodic changes across datasets spanning extended time intervals, we implemented an element-wise division approach that utilizes half of the same-group features from the CNNs to divide the others. This division process was evolved with inspiration from previous studies that considered oscillatory features within specific frequency bands of interest in relation to more general or stabilized signal features^15,28,31,32^. Both the original numerators and resulting divided outcomes, denoted as original and relative features, were concatenated in a way that both types of features are considered for further model training.
- Feature squeeze and excitation block: The feature squeeze and excitation block in our model is designed to rescale spectral features, emphasizing those with greater importance. As it is a widely known concept that rescaling features using an encoding and decoding approach such as squeeze and excitation networks or attention modules effectively highlight informative features^50,51^, the feature squeeze and excitation block of our model employs the similar concept. The outcome from the feature extraction block is processed in a way that the feature dimension is encoded and decoded back to its original shape for each length-dimension sample, with ReLU activation function used in between. Specifically, the oscillatory features are squeezed and excited for each temporal slice, with encoding and decoding parameters shared across temporal samples, resulting in an output of the same size as the shape before encoding. The resulting feature after further going through sigmoid activation was multiplied element-wise to the original output of the feature extraction block to rescale the features.
- Bi-directional LSTM block: A three-layer stacked bi-directional LSTM was employed in our model to consider temporal changes in the LFP features within the provided time window. Recent findings suggest that beta burst duration, in extension to elevated beta power, also closely correlates with motor impairment^18,23,31^. Our bi-directional LSTM block was thus used to consider temporal oscillatory changes over time.
- Regression block: The outcome after the bi-directional LSTM block was subsequently merged to finalize the regression model. By using a groupwise convolutional layer, features from the forward and backward LSTMs were first separately extracted. These features were then merged by sequentially going through convolutional and dense layers, and through ReLU activation function to complete our regression model that inputs 5-second LFP signals to output a single value weight shift prediction.

Note that our N2GNet did not utilize bias for the layers used in the model except for the layers in the feature squeeze and excitation block.

### Model training

The overall algorithm was implemented with Python and the deep learning model was designed using Pytorch. The NVIDIA GeForce RTX 4090 GPU was used to train the model, and the adaptive moment algorithm (ADAM) optimization was used with a learning rate of 1e-5. The l1 loss function was used to measure errors between predicted results and actual labels, and a batch size of 16 was used for the model training process. The model was trained with a maximum epoch iteration set to 2000, and the early stopping was held whenever the model did not improve their validation loss for 100 epochs. The trained model from the epoch with the least l1 error rates computed using the validation dataset was selected to evaluate with the testing dataset (Fig. 5b).

The model for our experiment takes the most recent 5-second LFP signals of a particular time point, derived directly from the band-pass filter, as an input to predict the weight shift over the last 2 seconds. The datasets for training, validation, and testing are formed in a way that they include the last 5-second LFP signals and a single label representing the last 2 seconds of weight shift at every 0.1 second stride within each task duration. The model was also trained and evaluated in a subject-dependent manner, where the model was trained, validated and tested separately on each participant.

### Evaluation and analysis

The length of data varied across tasks and the weight shifts performed by participants, which were rescaled within the range of 0 and 1 and used as dataset labels, were not uniformly distributed within each task. Taking into account such factors, both mean absolute error (MAE) and mean squared error (MSE), which are two commonly used evaluation metrics for assessing regression performance in machine learning models^29^, were computed on both validation and test datasets. This was done in order to provide more comprehensive insights into the performance of the models evaluated in this study, and also with consideration that both validation and test sets were obtained from visits that occurred considerably later than the visits from which the training data were collected. We also conducted correlation analysis using Kendall tau coefficient^52^, a non-parametric statistic to measure the association between the two variables, to quantify correlations between the model’s predicted results and the weight shifts. Correlations of each 2-second average beta power computed from the LFPs of the two leads with respect to the weight shifts were also quantified and compared with those between N2GNet’s predictions and the weight shifts to explore the benefits of using N2GNet over beta power.

A model ablation study, a commonly used analysis method for deep learning to investigate how each block composing the final model contributed to its performance^53,54^, was conducted with our N2GNet. A total of seven other models derived from our N2GNet were thus designed, assessed, and compared. The models were named as FExt-Div, FExt-Div+SE, FExt-Div+Bi, FExt-Div+SE+Bi, FExt, FExt+SE, and FExt+Bi, depending on which parts of the original N2GNet model were used or neglected.

To explore which frequency band signals had more impact on the trained model, we measured the variation ratio of the model’s output concerning different frequency band ranges. Specifically, the following procedure was conducted on each participant’s model to investigate which frequency bands had more influence over other bands:

- The LFP data from the training set was band-pass filtered with each frequency band of interest, and were inputted to the model.
- The model’s outcomes prior to the last ReLU activation from the regression block were retrieved for each frequency band of interest, and the variance of these outcomes from the same frequency band of interest were measured. Thus, a single measure was produced per a single frequency band of interest, representing the variance of its corresponding outputs.
- Variance measures from different frequency bands in a single participant were normalized as a variation ratio, such that the sum of all frequency bands equaled 1 for the participant.

With the models used in our study producing a single value per input, the variation ratio derived from the resulting values provides insights into which frequency band signals were more influential in producing the final outcome.

The variation ratios in our study were measured using six different frequency bands: delta and theta (≤8 Hz), alpha (8–13 Hz, inclusive), low-beta (13–20 Hz, inclusive), high-beta (20–36 Hz, inclusive), low-gamma (36–70 Hz, inclusive) and high-gamma (≥70 Hz).

### Statistical analysis

For statistical comparisons involving two groups with independent variables, we utilized the Mann–Whitney *U*-test. We also utilized the Wilcoxon signed-rank test for statistical comparisons that involved paired samples. A Kendall tau correlation coefficient analysis was used to quantify the association between two data measures. These statistical tests were performed considering that the number of participants is relatively small (*n* = 9 for both TD and AR groups, and a total of 18 PD participants), and the non-parametric tests do not assume normal distribution of the data. Despite the multiple comparisons, we reported measures with *p*-values above a threshold of 0.05 as not significant for Mann-Whitney U tests, taking a conservative approach to conclude that there was no significance in the comparisons between the two groups. A threshold of 0.025 (0.05/2) was considered significant for p-values from the Wilcoxon signed-rank tests considering a Bonferroni correction for the two tests conducted: one from the comparison with validation sets and the other from the comparison with test sets.

## Supplementary information

Supplementary information

## Data Availability

The datasets used for the current study are not publicly available, but may be available to qualified researchers from the corresponding author upon reasonable request.

## Code availability

The architectures of our N2GNet model and the other evaluated models are described in detail in the Methods section and figures. The codes for the study are not publicly available, but may be available to qualified researchers from the corresponding author upon reasonable request.
