# Supplementary material for: N2GNet tracks gait performance from subthalamic neural signals in Parkinson’s disease
Source: NPJ Digit Med. 2025 Jan 4;8:7. doi: 10.1038/s41746-024-01364-6 (PMC11700158; doi:10.1038/s41746-024-01364-6)
Supplement: Supplementary file 1 — Supplementary information [file 41746_2024_1364_MOESM1_ESM.pdf]

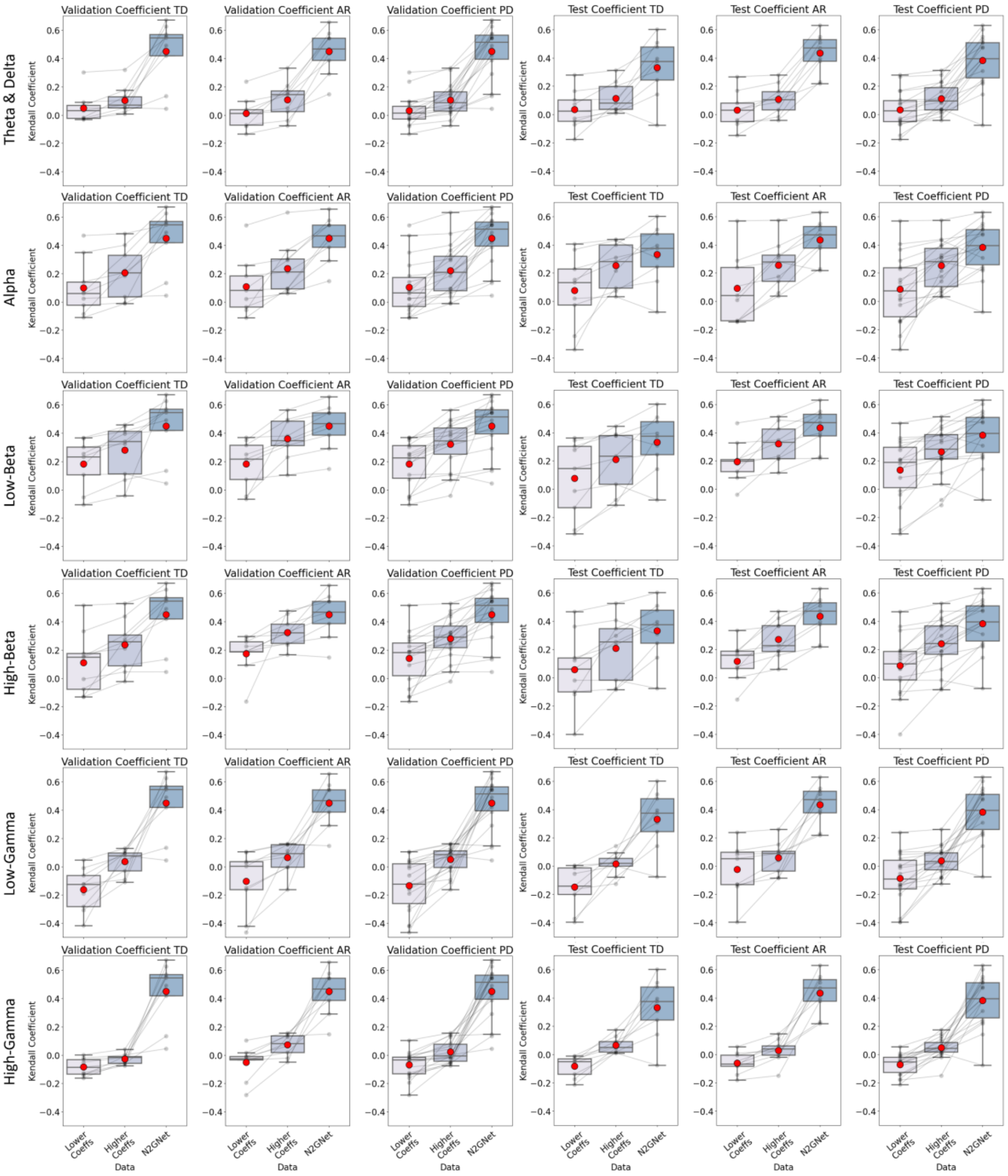

Supplementary Figure 1: Comparisons of correlation results from validation and test sets for TD group, AR group, and for all PD participants. A total of six other possible frequency bands of interest were additionally measured and compared with the results from our N2GNet model for reference. A positive Kendall coefficient with respect to the power measure of a specific frequency indicates that its suppression is associated with increasing weight shifts, while a negative Kendall coefficient represents that an increase in power correlates with increasing weight shifts. Thus, frequencies that are suppressed with respect to movement should refer more to the distributions of higher coefficients, whereas frequencies that increase with movement should focus on distributions of lower coefficients. The x-axis, from left to right, indicates the lower coefficients out of the two band powers measured from each participant, the higher coefficients of the two, and the coefficients computed with N2GNet results. The gray dots indicate correlation coefficients from each participant, and the dots in red represent the mean value. The boxplots represent first quartile, median, and third quartile for lower, middle, and upper lines in the boxes, respectively, whereas the whiskers represent 1.5 times the IQR extending above the first quartile and below the third quartile.

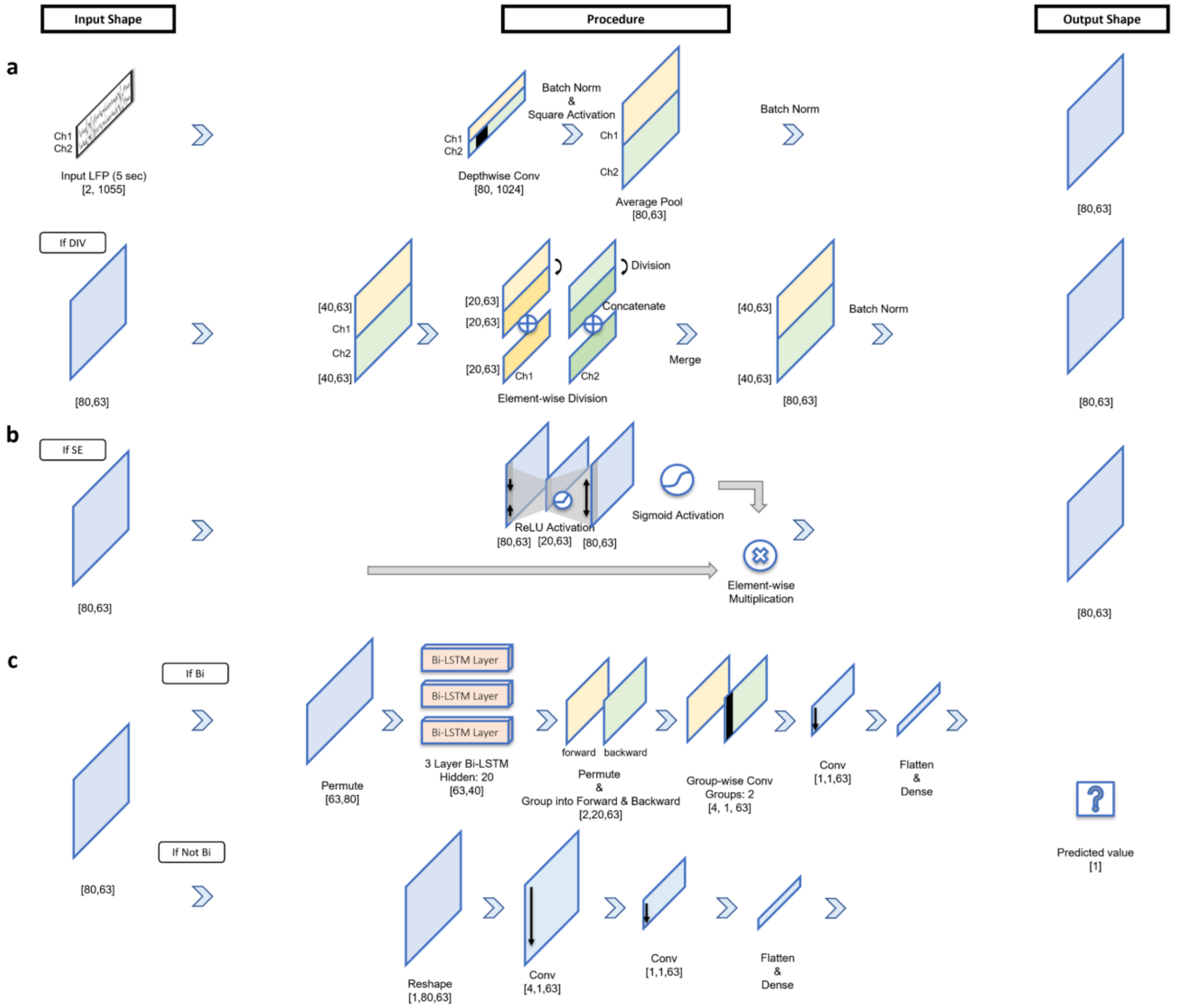

Supplementary Figure 2: A detailed flow of the models used in our model's ablation study. a) All the models used in our study first proceeded through feature extraction with the inputted LFP signals. The model underwent element-wise division if it included the division process (FExt), or it was neglected in models that did not include division (FExt-Div). b) For the models that contained the feature squeeze and excitation block as one of their components (+SE), the output from the feature extraction block was rescaled based on the features produced through the encoding and decoding layers. c) The resulted features finally went through either one of the two procedures: if the model considered bi-directional LSTM as its component (+Bi), the model sequentially proceeded through a 3-layer Bi-LSTM, a group-wise convolutional layer, and through convolutional and dense layers. If the model did not include bi-directional LSTM, the model proceeded through convolutional and dense layers. The ReLU activation was used to finalize regression that outputted a single value representing the weight shift.

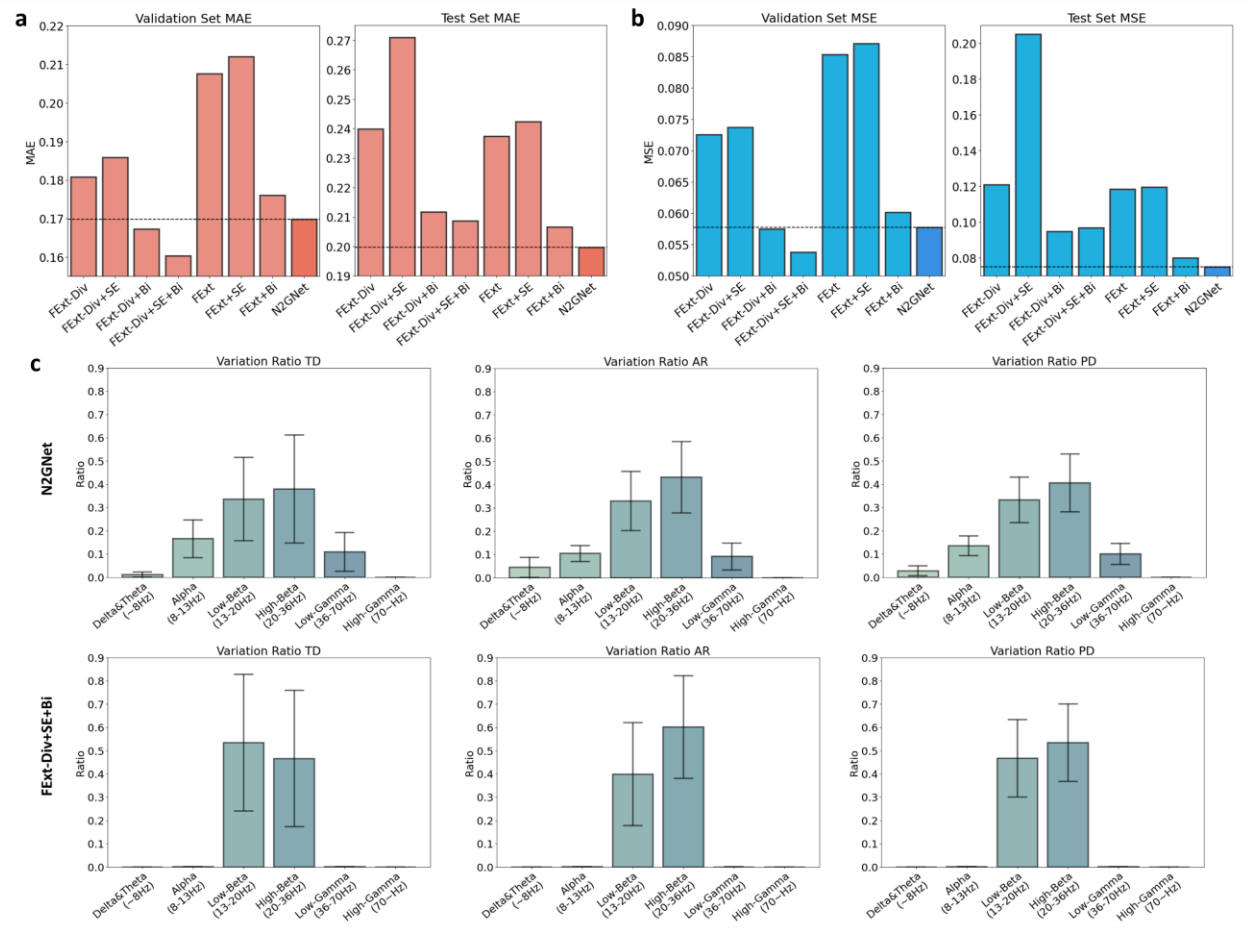

Supplementary Figure 3: Model ablation study results and analysis with models trained, validated and tested with beta band-pass filtered LFP signals (13-36 Hz). The average error rates using a) MAE and b) MSE were obtained from validation and test datasets for eight different model designs derived from our proposed N2GNet. c) Variation ratio results quantifying the impact the data of certain frequency bands had on the model's output. Both N2GNet and our proposed architecture without the element-wise division process from the feature extraction block (FExt-Div+SE+Bi) were analyzed to explore the effect of considering relative oscillatory features in our model. The error bars represent 95% confidence intervals.

Supplementary Table 1: MDS-UPDRS III scores for visits

Participants	Training Set Visit	Validation Set Visit	Test Set Visit
1	22	27	36
2	20	31	12
3	44	45	55
4	42	47	65
5	56	43	41
6	66	61	59
7	44	33	NaN
8	50	43	62
9	23	NaN	27
10	27	23	32
11	45	45	58
12	41	41	54
13	34	31	27
14	43	NaN	36
15	NaN	7	5
16	11	NaN	18
17	16	20	29
18	38	36	36

MDS-UPDRS III scores from participants in off medication and OFF stimulation, evaluated within three months of each visit.

NaN: the score has not been assessed.

MDS-UPDRS: Movement Disorder Society-Unified Parkinson’s Disease Rating Scale.
